# Supplementary material for: Naturally occurring ACE2 stalk variants are differentially released from the cell
Source: Sci Rep. 2026 Jul 8;16:21184. doi: 10.1038/s41598-026-60888-x (PMC13342293; doi:10.1038/s41598-026-60888-x)

Supplementary Figure S1:

(A) AP activity was measured in lysates from untransfected and wt ACE2-AP-transfected HEK293 cells. Untransfected cells showed no measurable endogenous AP activity, confirming that the AP signal detected in transfected cells originates from the expressed ACE2-AP constructs.

(B) uncropped blots from figure 1C.

(A)

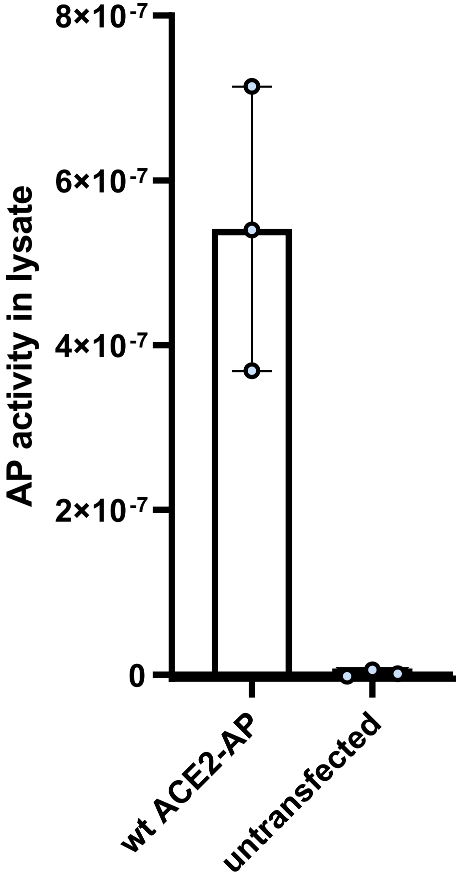

(B)

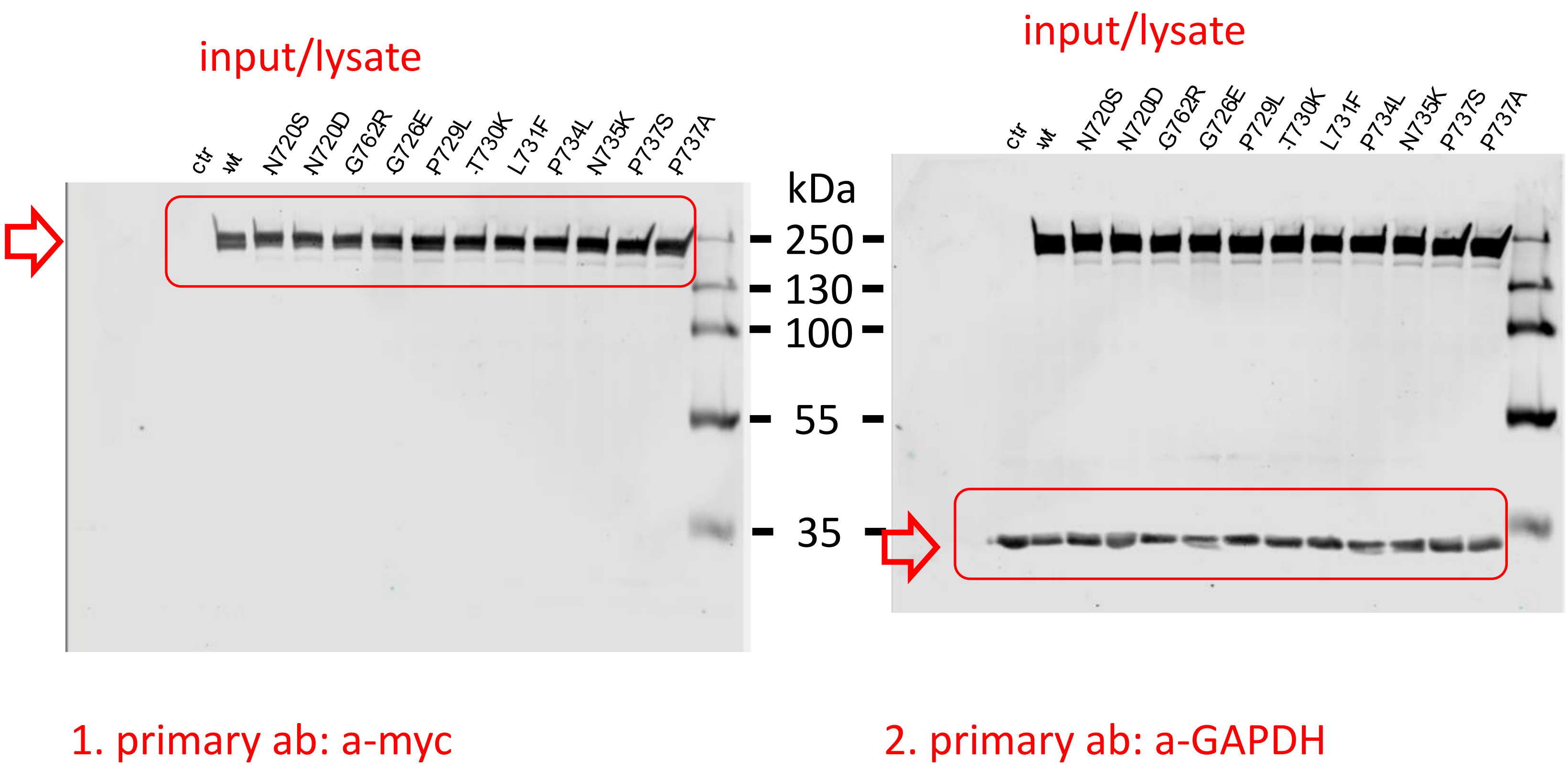

Supplementary Figure S2

Uncropped blots from figure 1F.

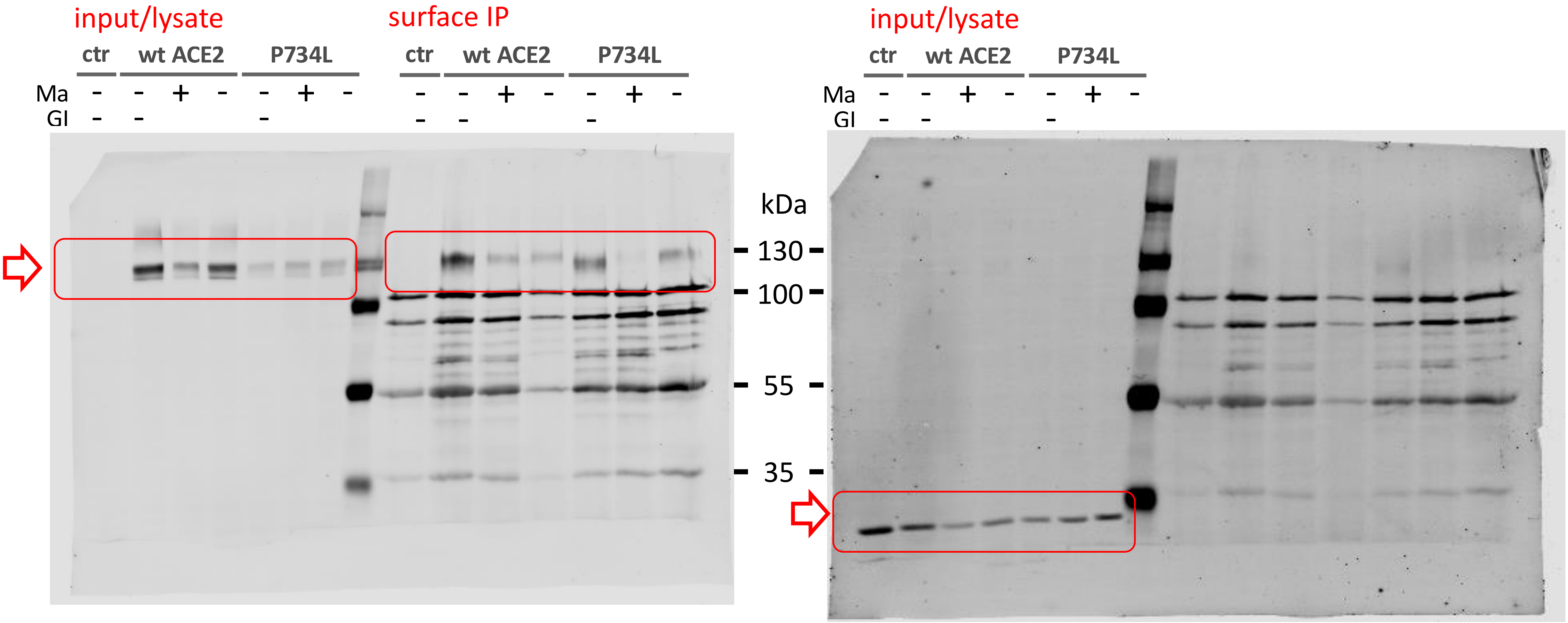

1. primary ab: a-myc

2. primary ab: a-GAPDH

Supplementary Figure S3

Uncropped blots from figure 2B.

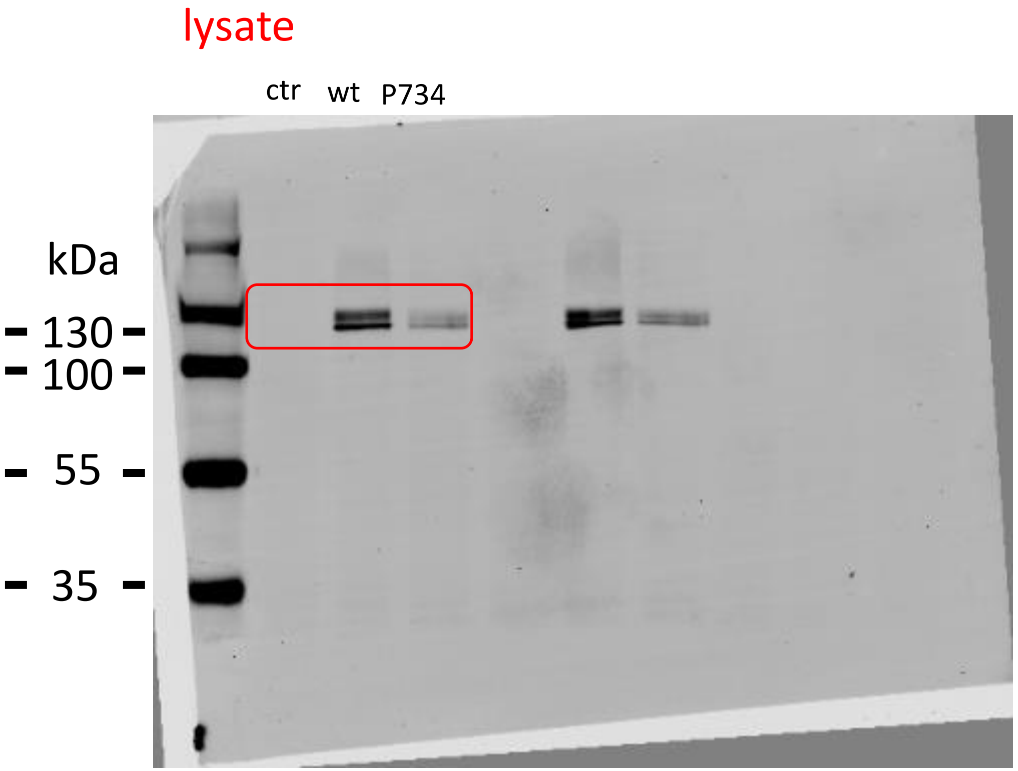

1. primary ab: a-myc

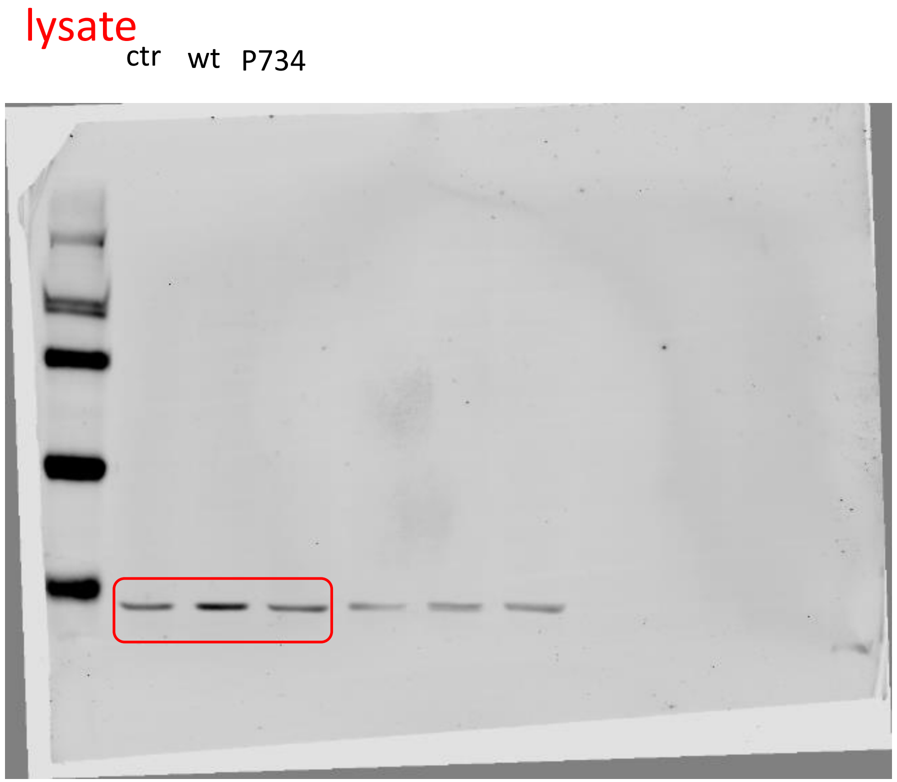

2. primary ab: a-GAPDH

Supplementary Figure S4

(A) Uncropped blots from figure 2F.

(B) Representative flow cytometric analysis (from figure 2G): HEK293 cells stably overexpressing wt ACE2, P734L and or GFP (control, ctr) were transiently co-transfected with HA-tagged B0AT1. HEK293 cells without HA-tagged B0AT1 transfection were used as negative control (neg).

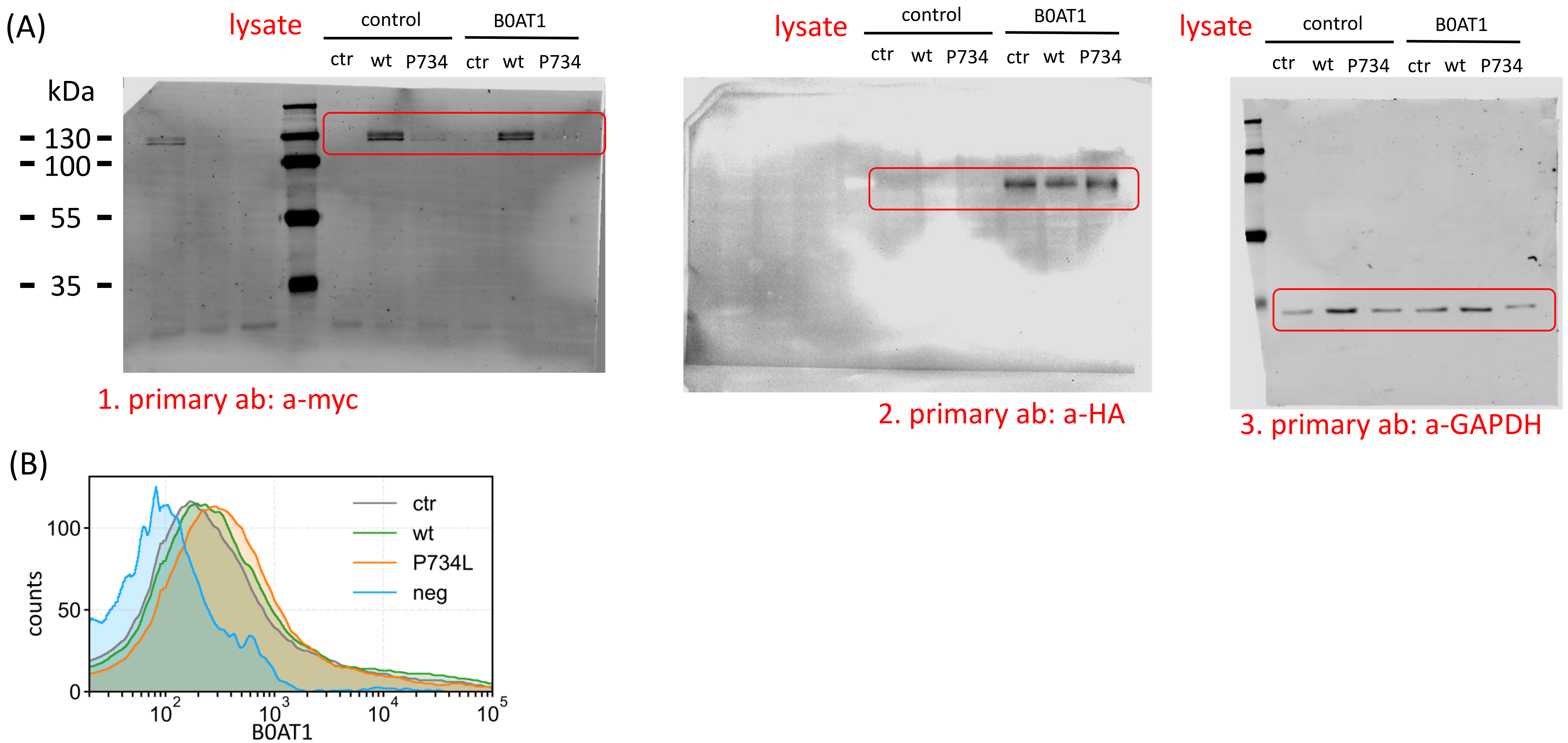

Supplementary Figure S5

Uncropped blots from figure 3B.

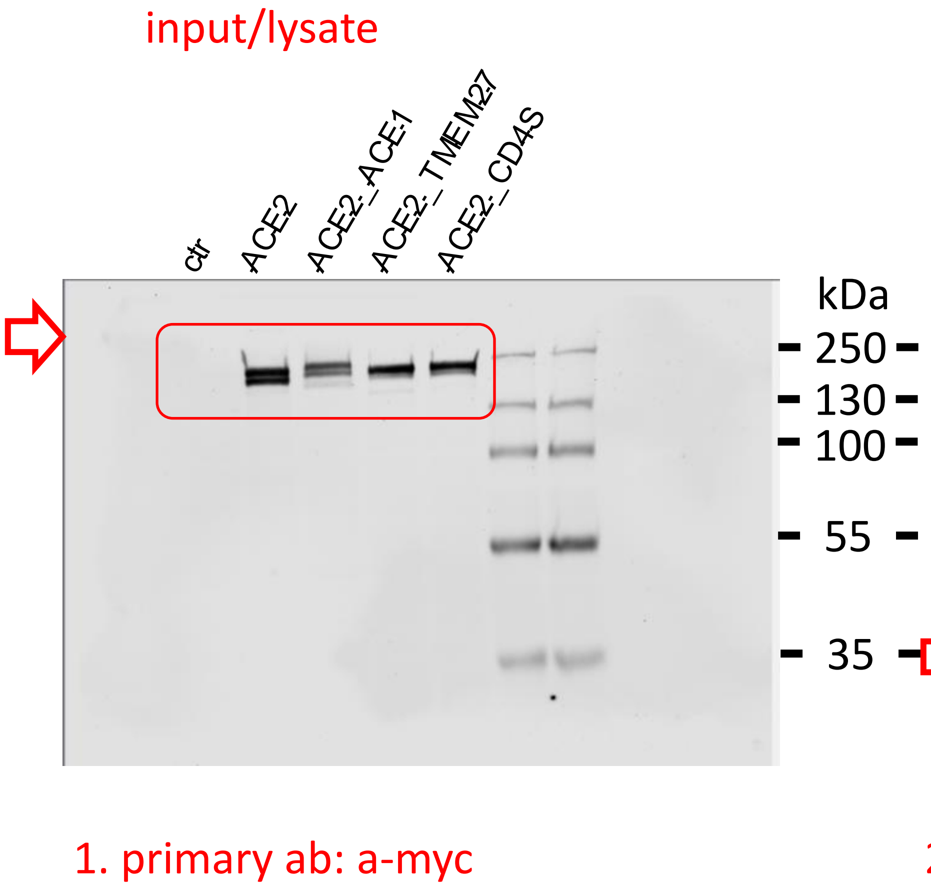

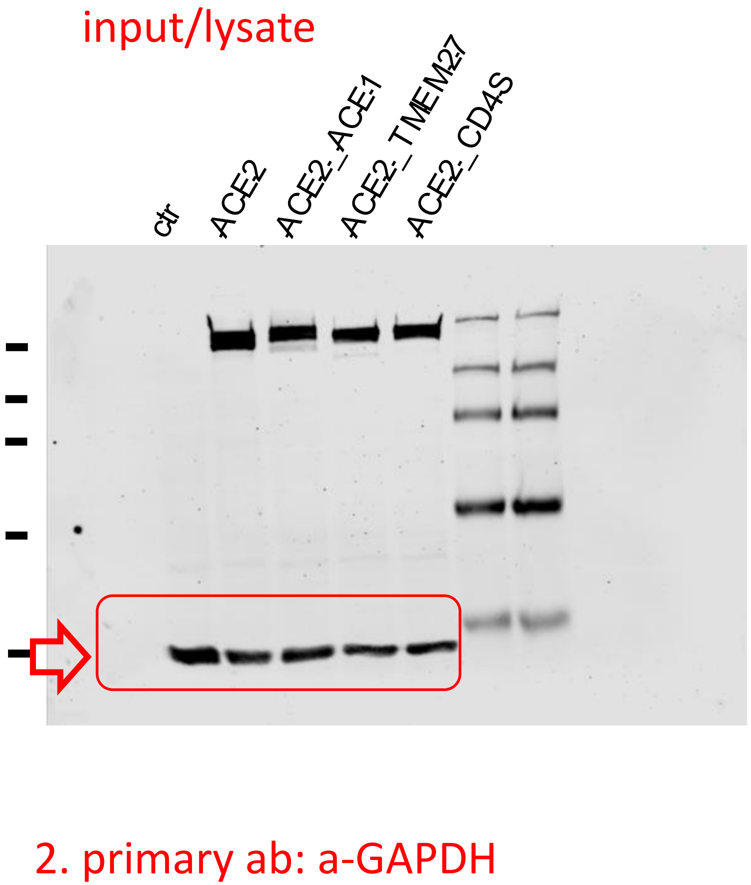

Supplement: Supplementary file 1 — Supplementary Information. [file 41598_2026_60888_MOESM1_ESM.pdf]
